# ESKAPE Pathogens in a Hungarian Emergency Department: A 5-Year Retrospective Observational Study of Prevalence and Resistance Patterns Utilizing the AWaRe Framework

**DOI:** 10.3390/antibiotics15090827

**Published:** 2026-08-25

**Authors:** Peter Erdelyi, Ria Benko, Laszlo Papp, Mario Gajdacs, Maria Matuz, Dorottya Gyarfas, Edit Hajdu, Laszlo Orosz, Adam Visnyovszki, Zoltan Peto

**Affiliations:** 1 Department of Emergency Medicine, Albert Szent-Györgyi Health Center, University of Szeged, 6725 Szeged, Hungary; erdelyi.peter@med.u-szeged.hu (P.E.); papp.laszlo@med.u-szeged.hu (L.P.); peto.zoltan@med.u-szeged.hu (Z.P.); 2 Doctoral School of Experimental and Preventive Medicine (KIMODI), Albert Szent-Györgyi Health Center, University of Szeged, 6720 Szeged, Hungary; 3 Institute of Clinical Pharmacy, Faculty of Pharmacy, University of Szeged, 6725 Szeged, Hungary; matuz.maria@szte.hu; 4 Institute of Clinical Pharmacy, Albert Szent-Györgyi Health Center, University of Szeged, 6725 Szeged, Hungary; 5 Department of Public Health, Albert Szent-Györgyi Medical School, University of Szeged, 6720 Szeged, Hungary; gajdacs.mario@med.u-szeged.hu; 6 MTA-SZTE Lendület “Momentum” Anthropogenic Stress and Plant Resilience Research Group, Közép fasor 52, 6726 Szeged, Hungary; 7 Albert Szent-Györgyi Faculty of Medicine, University of Szeged, 6720 Szeged, Hungary; gyarfas.dorka@gmail.com; 8 Infectious Disease Ward, Department of Internal Medicine, University of Szeged, 6720 Szeged, Hungary; horvathne.hajdu.edit@med.u-szeged.hu; 9 Department of Medical Microbiology, Albert Szent-Györgyi Health Center and Faculty of Medicine, University of Szeged, 6725 Szeged, Hungary; orosz.laszlo@med.u-szeged.hu; 10 Central Hospital of Southern Pest—National Institute of Hematology and Infectious Diseases, 1097 Budapest, Hungary; visnyovszki.adam@dpckorhaz.hu

**Keywords:** emergency medicine, antibiotic resistance, ESKAPE pathogens, surveillance, cumulative antibiogram, AWaRe classification

## Abstract

**Background/Objectives**: Surveillance of local antibiotic resistance plays a critical role in guiding empirical antibiotic therapies in emergency departments (EDs). This study aimed to collect and analyze a five-year period of prevalence and AMR rates among “ESKAPE” pathogens in a tertiary-care ED setting. **Methods**: This retrospective observational study included a complete census of microbiological specimens collected from patients presenting to the emergency department between 1 January 2019 and 31 December 2023. Data was retrieved from the MedBakter laboratory information system. Non relevant isolated and duplications were excluded. Only the first isolates per patient per phenotype were included in the final dataset. **Results**: The final dataset contained 6510 isolates. The most frequent isolates were *E. coli* (2717, 41.7%), *K. pneumoniae* (715, 11.0%), and *P. mirabilis* (696, 10.7%) followed by *E. faecalis* (662, 10.2%). Prevalence of multi-drug resistance (MDR) was 23.26% among “ESKAPE” pathogens, with highest prevalence in *M. morganii* (79.6%), *P. stuartii* (78.5%) and *S. marcescens* (77.1%). Difficult-to-treat resistance (DTR) was found in 32 isolates, while extensive drug resistance (XDR) was found in 5 isolates. The prevalence of other high-priority MDR bacteria was 12.4% for MRSA and 4.7% for VRE, while among Gram-negative bacteria the prevalence of carbapenem-resistant Enterobacterales was 0.52%. The prevalence of cephalosporin-resistant Enterobacterales varied greatly between 0 and 33%. **Conclusions**: Despite these important AMR trends, the cumulative antibiogram, according to the World Health Organization (WHO)-issued Access, Watch and Reserve (AWaRe) classification, revealed room for empirical antibiotic choices that spare Watch and Reserve agents. The results will aid the revision of the local antibiotic guidelines.

## 1. Introduction

Antimicrobial resistance (AMR) has become a critical public health concern globally [1]. Based on the most recent estimates, bacterial AMR was directly attributable to 1.14 million deaths and was further associated with 4.71 million deaths in 2021 [2]. Furthermore, if current trends persist, the mortality linked to AMR is expected to increase sharply (i.e., attributable mortality to 1.91 million, associated mortality to 8.22 million, annually by 2050), leading to around three deaths every minute [2]. The management of AMR is often associated with treatment failure, extended hospital stays, and the necessity of using “last resort/reserve” antimicrobial drugs with high expenditures and worse adverse effect profiles [3]. Subsequently, AMR is also responsible for a notable excess financial burden (vs. treating susceptible infections), ranging from approximately USD 2500 to USD 30,000 per patient episode. According to the estimations of the World Bank, AMR may be responsible for an annual global GDP loss of USD 1 trillion by 2030, and in a worst-case scenario, this figure may scale up to a global USD 3.4 trillion GDP loss per year by 2050 [4]. Beyond the naturally occurring development of AMR (i.e., “selection pressure”), numerous drivers have been implicated in accelerating the AMR crisis. The misuse and overuse of antibiotics in the human and veterinary care sectors is one of the most important drivers of AMR. According to the World Health Organization (WHO), the current efficacy of antibiotic research and development (R&D) pipelines is inadequate to tackle elevated levels of AMR in pathogenic bacteria of interest [5,6]. Therefore, together with reinvigorating the antibacterial R&D landscapes, policies to address AMR should prioritize infection prevention and control measures to decrease the incidence of infectious diseases (especially within healthcare settings), to enable universal access to suitable diagnostic tools and AMR surveillance data within the local context, and to reduce the rate of irrational antibacterial prescribing. While evidence on the global trends of AMR is crucial to inform global health policy initiatives, data originating from local mapping of resistance trends facilitate the tailoring of empirical antibiotic treatment guidelines [7]. The usefulness of cumulative antibiograms—either in an institutional or in a narrow geographical context—has been demonstrated by earlier studies [8]. However, given that the emergence and alterations in AMR trends are a dynamic and continuous process, these antibiograms must undergo periodic reassessment.

As the primary interface between the community and the hospital environment, emergency departments (EDs) serve as critical hubs for the surveillance and management of AMR [9]. EDs are a key destination for patients to receive treatment for severe, community-acquired infections that have been missed or failed to be managed in outpatient settings. Therefore, EDs may be considered settings where more severe infections are concentrated with higher representation of resistant pathogens [10]. As a consequence, surveillance of clinically relevant microbial isolates detected in ED patients may provide special insight into the prevalence of AMR within the community. As antimicrobial treatment decisions in the emergency care setting are made almost exclusively on an empirical basis, the strategic choice of antimicrobial agents is paramount.

“ESKAPE” pathogens are often considered some of the most concerning bacterial pathogens in clinical practice, characterized by a heightened rate of resistance towards both frequently used and last-resort antimicrobials, commonly causing severe, life-threatening infections in the most vulnerable patient populations [11]. The importance of “ESKAPE” pathogens has been best depicted by the updated WHO Bacterial Priority Pathogens List from 2024 [12], where antibiotic-resistant representatives of the “ESKAPE” group represented the whole “critical priority” and most of the “high priority” groups. Despite the pivotal role of EDs in the management of common infections, published ED-specific AMR data are notably scarce or have a narrow focus (i.e., they only include bloodstream infections [13,14,15] or urinary tract infections, or they focus on only one specific pathogen) [16,17,18]. By responding to the WHO’s call for continuous surveillance—outlined in the Global Action Plan on Antimicrobial Resistance (GAP-AMR)—the present study aimed to collect and analyze a five-year period (2019–2023) of prevalence and AMR rates among “ESKAPE” pathogens in a tertiary-care ED setting. The results are intended to optimize institutional antimicrobial stewardship interventions locally, while providing local, longitudinal, setting-specific data on AMR.

## 2. Results

### 2.1. Descriptive Results

**Figure 1:**
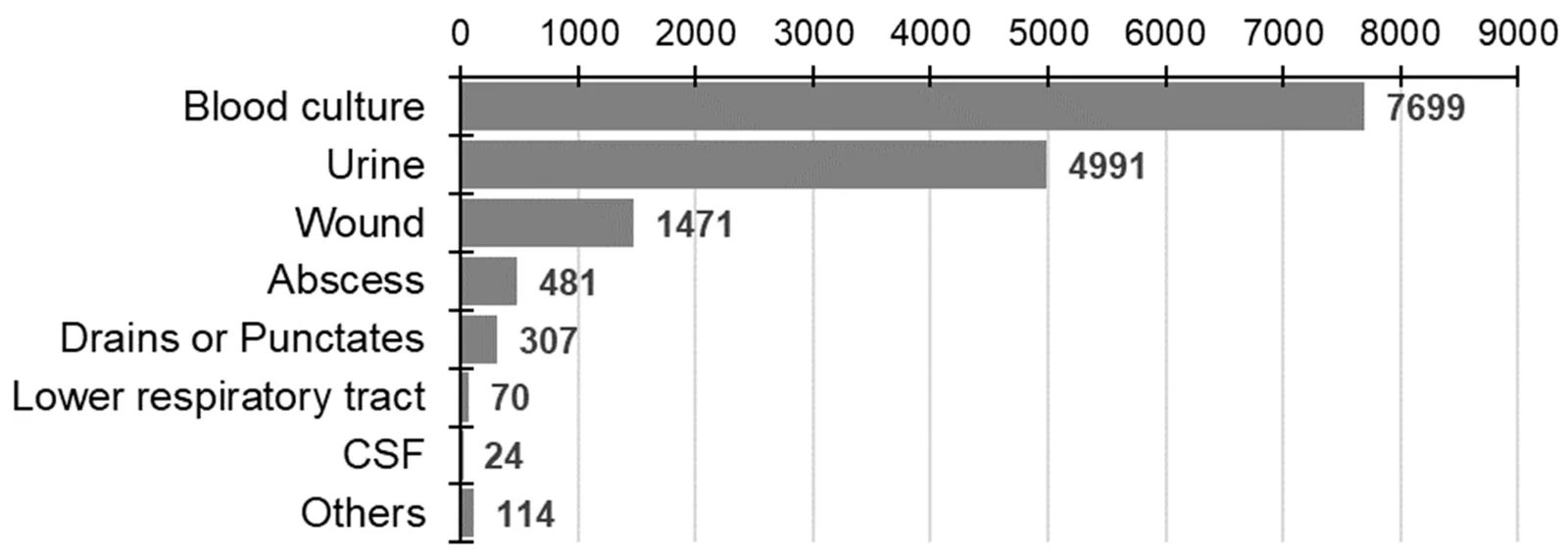
Number of and sample type distribution of all positive microbiological isolates from the ED (before data deduplication) during the study period (2019–2023).

During the 5-year study period, a total of *N* = 15,157 positive isolates—sent from the ED—were detected; the distribution of sample types is shown in Figure 1.

**Figure 2:**
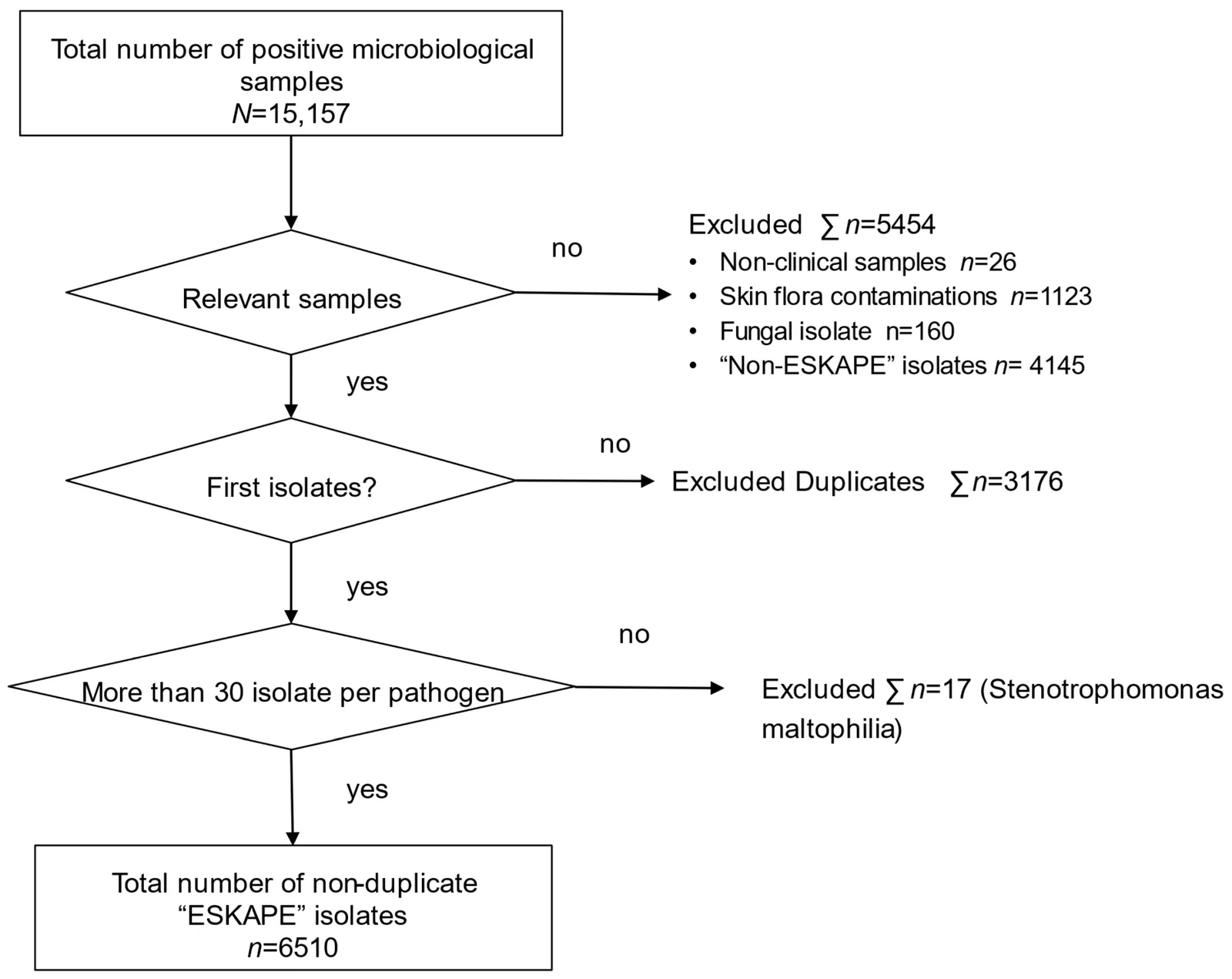
Flowchart describing the data selection process corresponding to relevant “ESKAPE” isolates.

Based on our previously determined inclusion and exclusion criteria, and the operational definition used for “ESKAPE” pathogens (i.e., including *Enterococcus* spp., *S. aureus*, *S. maltophilia*, *Klebsiella* spp., *A. baumannii*, *P. aeruginosa* and other relevant members of the Enterobacterales order), non-clinical (screening) samples (e.g., throat or vulvar swabs; *n* = 26), samples associated with contaminants from skin flora (*n* = 1123), and samples containing fungal isolates only (*n* = 160) were excluded. Non-ESKAPE isolates were also excluded (*n =* 4145). As a next step, duplicates were excluded (*n* = 3176), including only the first isolates per patient per phenotype for each species across the entire study period. Finally, *Stenotrophomonas maltophilia* was excluded from the final dataset due to an insufficient sample size (*n* = 17) (Figure 2). In the final dataset, a total of *n* = 6510 non-duplicate, clinically relevant “ESKAPE” isolates were included, which were subject to further analysis, as shown in Table 1.

Gram-negative bacteria represented the majority of the isolates (*n* = 5231, 80.4%), among which *E. coli* was responsible for more than 40% of the cases, followed by *Klebsiella pneumoniae*. (*n* = 715, 11.0%) and *Proteus mirabilis* (*n* = 696, 10.7%), respectively. Gram-positive bacteria were detected in 1279 cases (19.6%) (Table 1).

### 2.2. Antibacterial Resistance Rates

All the detected microorganisms were classified based on their resistance rates to the relevant antibacterials tested, which have been summarized in Table 2. In total, *n* = 2479 (38.1%) of the isolates did not show detectable resistance beyond intrinsic resistance (i.e., wild-type microorganisms), while in a further *n* = 4031 (61.9%) isolates, usual drug resistance (UDR) was detected. Among UDR isolates, *n* = 1514 isolates (23.3%) were proved to have a multi-drug resistant (MDR) phenotype, with high levels of disparities among different species: the highest prevalence of MDR strains was noted among *M. morganii*, *P. stuartii* and *S. marcescens*, followed by *E. faecium* and *A. baumannii* (79.6%, 78.5%, and 77.1%, followed by 75.0% and 70.7%, respectively). Difficult-to-treat resistance (DTR) was identified in *n* = 32 cases, most detected in *A. baumannii*, where 23 specimens (56.1%) showed the DTR phenotype. Furthermore, a total of five extensively drug-resistant (XDR) bacteria were identified (two among *A. baumannii* and three among *K. pneumoniae*); among these, three cases showed susceptibility only to cefiderocol. Pan-drug-resistant (PDR) isolates were not identified (Table 2).

### 2.3. Cumulative Antibiograms Corresponding to “ESKAPE” Pathogens

The cumulative antibiograms (i.e., rates of antibiotic susceptibility for respective isolates, %), corresponding to “ESKAPE” pathogens included in our analysis, are shown listed according to the 2025 WHO Access, Watch, Reserve (AWaRe) categories, displayed in Table 3, Table 4 and Table 5. For instances where the number of tested isolates for a specific antimicrobial agent was under 30 (*n* < 30), antimicrobial susceptibility is reported as absolute counts rather than percentages.

Within the "Access" group of antibiotics, many Gram-negative strains showed less than 80% sensitivity to amoxicillin/clavulanic acid. The important exceptions were *K. oxytoca*, *K. varicola*, *E. coli*, and *Salmonella* spp. Notably, *E. coli*—the most frequently identified isolate—showed 83.0% susceptibility, making it a viable option for treating non-severe infections empirically, where *E. coli* is suspected. Also, *E. coli* demonstrated almost complete (98.0%) sensitivity for nitrofurantoin, and aminoglycosides, but had decreased sensitivity (75.4%) to sulfamethoxazole/trimethoprim. Gram-negative isolates showed predominantly over 90% susceptibility to amikacin, apart from *A. baumannii* and *P. mirabilis*. Gentamicin susceptibility was decreased below 80%, in cases of *M. morganii*, and *P. mirabilis*. *A. baumannii* showed markedly low rates of susceptibility against all Access group antibiotics. Among Gram-positive strains, methicillin-susceptible *S. aureus* (MSSA) had over 90% susceptibility for all “Access” antibiotics, except for clindamycin (82.8%). Methicillin-resistant *S. aureus* (MRSA) had completely retained susceptibility for doxycycline and sulfamethoxazole/trimethoprim and 90.6% susceptibility for gentamicin. *E. faecalis* had intact susceptibility for aminopenicillins (100.0%) and nitrofurantoin (99.3%), with a notably decreased susceptibility against gentamicin (66.6%). *E. faecium* had low rates of susceptibility to all “Access” group antibiotics (Table 3).

Within the context of “Watch” group antibiotics, Gram-negative strains generally showed an almost uniform (100.0%) susceptibility to carbapenems, with exceptions seen among non-fermenters, i.e., in *P. aeruginosa* (87.3%) and *A. baumannii* (43.9%). Among Enterobacterales, carbapenem resistance was 0.52%, while cephalosporin resistance varied between 0% and 33%. *E. coli* had over 85.0% susceptibility to all antibiotics except for fluoroquinolones (~76%). *K. pneumoniae* had over 80% susceptibility only to tobramycin and carbapenems. Other *Klebsiella* species generally showed over 90% susceptibility to all “Watch” group antibiotics, except for *K. aerogenes* (previously *E. aerogenes*) against third- and fourth-generation cephalosporins and piperacillin/tazobactam. *P. mirabilis* had a susceptibility rate of less than 75% to all “Watch” group antibiotics except carbapenems. *P. vulgaris*, *Salmonella enteridis* and *S. marcescens* generally had over 90% susceptibility to all “Watch” group antibiotics except *Salmonella enteridis* for ciprofloxacin and *Proteus vulgaris* for cefixime. However, these figures warrant cautious interpretation due to the markedly low number of isolates evaluated. *P. aeruginosa* had over 85% susceptibility for piperacillin/tazobactam, ciprofloxacin and anti-pseudomonal carbapenems, and around 90% susceptibility for anti-pseudomonal cephalosporins, and over 95% for tobramycin. *A. baumannii* had a susceptibility rate of <45% to any “Watch” group antibiotics. Among Gram-positive strains, MSSA generally had over 95% susceptibility to all Watch group antibiotics apart from macrolides (81.5%). MRSA had completely retained susceptibility to glycopeptides and fusidic acid, and 98.4% susceptibility to rifampicin. *E. faecalis* had nearly 100% susceptibility to glycopeptides, imipenem and piperacillin/tazobactam, but had around 63% susceptibility to fluoroquinolones. The prevalence of vancomycin-resistant enterococci (VRE) during the study period was found to be 4.7% (among *E. faecium*, vancomycin resistance was present in 33.0% of isolates) (see Table 4).

Susceptibility rates for the “Reserve” group antibiotics were generally above 95% in the tested “ESKAPE” isolates, with very few exceptions. In the Gram-negative group, *P. mirabilis* also showed 87.5% susceptibility to ceftazidime/avibactam and 81.0% for ceftolozan/tazobactam. The prevalence of colistin-resistant *A. baumannii* and colistin-resistant *K. pneumoniae* was 2/26 and 1/20 cases, respectively. Results of cefiderocol and colistin susceptibility should be interpreted cautiously due to the low number of isolates evaluated. In the Gram-positive group, linezolid and tigecycline resistance was not detected, and almost completely retained quinpristin-dalfopristin susceptibility was observed (Table 5).

## 3. Discussion

The European Antimicrobial Resistance Surveillance Network (EARS-Net) [19] and the recently launched microbiological surveillance dashboard (JAMREYE) [20] provide large-scale European susceptibility trends, but they lack the necessary granularity needed for clinical decision-making at an individual department level. The WHO promotes the implementation of effective antimicrobial stewardship programs, relying on regularly updated local antibiograms as a primary tool for guiding empiric antibiotic therapy [21]. From 2004, providing inpatient AMR data is mandatory in Hungary; however, data regarding patients treated in the emergency setting are scarce. In addition, in the international literature, ED-specific resistance data are published mainly for certain microorganisms [17] or specific microbiological samples. ED-related cumulative antibiograms are rarely described [22,23,24], representing a knowledge gap despite the EDs’ critical role in early infection management. Addressing this gap might help in enabling emergency physicians with effective antibiotic choicesand the narrowest possible spectrum in a high-pressure environment where decision fatigue has been shown to adversely affect appropriate antibiotic prescribing patterns [25]. Our approach integrates local antimicrobial resistance patterns with international stewardship strategies, specifically the use of the WHO AWaRe (Access, Watch, Reserve) classification. Introduced in 2017, and updated biennially, the AWaRe framework categorizes antibiotics based on their clinical utility and their potential to drive resistance in real-world circumstances, providing a standardized tool for optimizing empiric therapy [26,27]. In alignment with target benchmarks established by the World Health Organization (WHO) and the United Nations (UN)—which specify that Access group agents should comprise at least 70% of total antibiotic consumption by 2030—our data were categorized according to the AWaRe framework to explore the feasibility of achieving compliance with these targets. Nonetheless, the primary objective of this study was to provide only a foundation for updating local empirical antibiotic guidelines. Consequently, these findings should not be translated directly into definitive clinical protocols, as only generalconclusions can be drawn. The possible antibiotic recommendations discussed in subsequent sections should therefore be interpreted within this cautious context.

To guide the upcoming revision of our clinical practice guidelines and contextualize the AST results (Table 3, Table 4 and Table 5), we categorized antimicrobial susceptibility into a three-tier system: ≥90% (green), 80–89% (yellow), and <80% (red). From a practical implementation standpoint, a susceptibility rate exceeding 90% supports the empirical use of an agent, even in severe infections. Rates between 80% and 89% remain acceptable for empirical therapy in non-severe infections. Susceptibility rates below 80% are considered inadequate for empirical use.

Although universally standardized susceptibility thresholds are lacking in the literature, our chosen cutoffs align with established clinical survey data and international guidelines. For instance, the empirical survey literature [28] indicates that clinicians typically demand a minimum of 90% coverage for severe, high-mortality scenarios and an 80% threshold for non-severe presentations. This is further reinforced by disease-specific recommendations, such as the IDSA uncomplicated UTI guidelines, which establish an 80% susceptibility cut-off for empirical regimen selection [29].

In our study, “ESKAPE” pathogens represented almost three-quarters (72.7%) of all positive bacterial isolates, among which Gram-negative bacteria were represented in 4/5 cases. In the WHO’s Bacterial Priority Pathogens List, carbapenem-resistant and third-generation cephalosporin-resistant Enterobacterales are considered critical priorities. In our cohort, carbapenem-resistant Enterobacterales occurred only sporadically (0.52%). In contrast, resistance to third-generation cephalosporins was notably more prevalent, as susceptibility rates varied between 67 and 100% in our study period. The most common pathogen was *E. coli*, which preserved its susceptibility to multiple “Access” antibiotics, such as nitrofurantoin and aminoglycosides. Nitrofurantoin was proven to be an effective first-line agent against *E. coli* in uncomplicated UTIs in recent studies [30,31,32,33] from different geographical locations (US, UK and Australia). Recently, Llor et al. found that nitrofurantoin was the most effective treatment in non-complicated UTIs when compared to fosfomycin (single and double dose) and pivmecillinam [34]. Nitrofurantoin should be considered as a first-line agent for lower UTI cases and aminoglycosides for upper UTI cases, where MDR is not expected. In contrast, *E. coli* showed notably decreased (below 80%) susceptibility to sulfamethoxazole/trimethoprim. In the Hungarian national surveillance registry [35], the susceptibility rate of sulfamethoxazole/trimethoprim against *E. coli* was identical to that in our findings at 75.3%. These findings are also consistent when compared with a multicenter study from 12 different EDs in the US, which investigated urine cultures positive for *E. coli* in 2022, where the rate of sulfamethoxazole/trimethoprim susceptibility was 74.9% [16]. That suggests that sulfamethoxazole/trimethoprim has no role in the empiric treatment of lower urinary tract infections in Hungary. The amoxicillin-clavulanic acid susceptibility rate was retained (83.0%) for *E. coli*, which is notably higher than the same-period national average of 76.2% [36]; therefore, even empiric use of amoxicillin-clavulanic acid might be considered in *E. coli*-related infections where it is applicable, enabling the increased reliance on the “Access” class of antibiotics.

Due to previous results (as susceptibility rates for ciprofloxacin stood at 67.9% from June 2014 to July 2019) during the current study period [37], a general fluoroquinolone-sparing antimicrobial guidance was in effect. Although a notable local improvement was observed in *E. coli* susceptibility to ciprofloxacin—in the current 2019–2023 period, susceptibility rose to 77.6%—fluoroquinolone restrictions are still warranted. This current result aligns with both the Hungarian national average (79.3%) [36] and the EARS-Net fluoroquinolone susceptibility European average (75.6%) [19] for the same period. Fluoroquinolone susceptibility also decreased below 80% for *K. pneumoniae*, *P. mirabilis*, *P. stuartii*, *A. baumannii*, MRSA, and *Enterococcus* spp. Conversely, *M. morganii* and *P. aeruginosa* displayed susceptibility rates of 84.3% and 87.0%, respectively, while all remaining species exhibited susceptibility exceeding 90%. These results support the continued use of a fluoroquinolone-sparing approach within our empiric antibiotic strategy. This approach is further substantiated by a ten-year surveillance study in the US, which demonstrated that restricting the use of fluoroquinolones may lead to an increase in the rate of susceptible isolates. Beyond the scope of AMR, the European Medicines Agency (EMA) also continues to advocate for restricted use of fluoroquinolones due to the risk of hazardous side effects [38]. These serious adverse reactions mainly impact the musculoskeletal, vascular and nervous systems, further justifying a cautious prescribing mandate in emergency medicine.

The *Klebsiella* genus represented the second largest isolate group within the Gram-negative bacteria. *K. pneumoniae* had susceptibility of below 80% to all “Access” and “Watch” group antibiotics, except for aminoglycosides and carbapenems, while the susceptibility to reserve antibiotics was preserved. During the study period, only two (0.2%) carbapenem-resistant *K. pneumoniae* (CRKP) isolates were found. Regardless, further surveillance of CRKP is imperative, due to the concerningly high prevalence in the neighboring Balkan countries—the highest prevalence was detected in Greece, reaching up to 46% in 2007 [39]. *P. mirabilis* was the third most frequent isolate, with a susceptibility rate above 90% only to carbapenems, greatly narrowing down its empirical treatment options. *M. morganii* showed the highest proportion of MDR strains (almost 80%) and retained a high (over 90%) susceptibility rate only to carbapenems and reserve antibiotics, and an over 80% susceptibility rate to fluoroquinolones, most cephalosporins and amikacin, providing multiple empirical antibiotic choices in non-severe *Morganella morganii* infections. In a European multicenter study from 2020 to 2022, the susceptibility rates for *M. morganii* toward third-generation cephalosporins and for carbapenems were similar (80.2% and 96.7%, respectively) [40].

*P. aeruginosa* was the sixth most common isolate. Among the “Access” antibiotics, amikacin sensitivity was 97.5%, while among “Watch” class antibiotics anti-pseudomonal cephalosporin susceptibility was also to be around 90%, allowing for the use of carbapenem-sparing strategies against pseudomonal infections [41].

Although community-acquired *A. baumannii* infections are rare [42]—in this study only *n* = 41 cases were identified—this group had the highest proportion of DTR isolates (23/41), meaning that carbapenem-resistant *A. baumannii*, one of the highest priority pathogens according to WHO, accounted for 56.1% of the *A. baumannii* cases. Colistin-resistant isolates were detected in two cases. Lodis et al. published an article on the burden of *A. baumannii* in the US between 2018 and 2022, where they reported that 80% of the carbapenem-resistant *A. baumannii* cases originated from emergency admissions [43].

When comparing our previous [37] and current surveillance studies, the most notable increases in susceptibility were observed within the “Access” group, especially for amikacin. In cases of *K. pneumoniae*, we detected a rise from 79.3% to 97.3%, in *E. coli* from 89.2% to 96.5%, and in *P. stuartii* from 36.4% to 100.0%. The observed changes can likely be attributed to recent updates in the EUCAST clinical breakpoints and interpretative criteria. According to the new terminology, the group formerly called “Intermediate” (originally considered non-susceptible) was redefined in 2019 as “Susceptible, increased exposure”, considering the therapeutic option of a higher-dose antimicrobial therapy that is still effective against the given microorganism; therefore, these microorganisms are now considered to be susceptible to the examined antimicrobials [44]. *M. morganii* showed a generally decreasing susceptibility trend for “Watch” group cephalosporins in the recent results, but no other obvious change was observed in the “Watch” and the “Reserve” group.

Gram-positive isolates represented approximately one-fifth of isolates in the final dataset. *E. faecalis* maintained over 90% susceptibility to aminopenicillins and glycopeptides, consistent with current national surveillance trends [35]. However, while the 5-year European EARS-Net data show a notable decrease in high-level gentamicin susceptibility for invasive *E. faecalis* (72.58%), our local susceptibility rate was even lower at 66.6% from invasive and non-invasive samples combined, in the same period. This difference is a serious cause for concern since high-dose aminoglycosides have an important synergistic role in treating *E. faecalis* infections (i.e., endocarditis) in combination with beta-lactams [45].

Globally, the prevalence of vancomycin-resistant *E. faecium* varies widely, ranging from 1% to 55% [46]. In our study, *E. faecium* demonstrated preserved full susceptibility only to “Reserve” group antibiotics, while 33.0% of isolates exhibited vancomycin resistance. While this is lower than the 41.6% resistance rate from the average 5-year Hungarian national surveillance data, both figures are deeply concerning when compared to the EARS-Net 5-year average, where only 20.14% of invasive isolates were vancomycin-resistant in Europe. *VRE* is an increasing threat to our healthcare system, and the efficacy of countermeasures is ambiguous. In a recent study, the prevalence of *VRE* did not change significantly, even with the introduction of targeted infection prevention and control (IPC) and antimicrobial stewardship program (ASP) measures [47].

The prevalence of MRSA was 12.4% among *S. aureus* during this study, which is slightly higher than the 5-year Hungarian average data from outpatient samples (11.14%) [35] but lower than the findings of a European surveillance study, where the average EU prevalence of MRSA was found to be 15.8% among invasive isolates (highest in Italy and Greece, with 29% and 27.3%, respectively) [48]. Given the low prevalence of MRSA in our findings, routine empiric MRSA coverage may not be routinely indicated. This allows for the implementation of vancomycin-sparing strategies, reducing unnecessary exposure to a WHO-defined “Watch” group antibiotic class.

### Strengths and Limitations

This study has several strengths and limitations that warrant consideration. In this study, results were analyzed independently of patient-level clinical data, and the findings were not correlated with specific clinical outcomes; therefore, causality between real-world clinical outcomes and the MDR phenotype in this setting was not studied. Although using an aggregate five-year dataset provides a robust overview of the period, it lacks annual granularity and may therefore mask subtle yearly fluctuations in susceptibility patterns, which would otherwise be notable, especially due to the COVID-19 pandemic. 

A further limitation involves the comparison between our current dataset (2019–2023) and our previous 5-year surveillance data (June 2014–July 2019). First, in 2019, EUCAST redefined the “Intermediate” category to “Susceptible, increased exposure” (I). Under the updated definitions, both “S” and “I” categories are classified as susceptible, whereas “I” results were formerly grouped with resistant isolates in our previous analyses, potentially influencing reported resistance trends [49]. Second, there is a minor chronological overlap between the two study periods (1 January to 31 July 2019), requiring caution when interpreting longitudinal trends. Third, sample selection in the present study adhered to an exclusion protocol based on the “first-isolate” principle, whereby duplicate isolates with the same phenotype from individual patients were systematically removed throughout the study duration. While this approach restricts direct comparability with our earlier surveillance data, it markedly strengthens the overall validity and reproducibility of our results. A final limitation of this study is that microbiological data were analyzed in aggregate without stratification by specific anatomical infection site (e.g., urine vs. blood vs. wound isolates) that might hinder more concrete, diagnosis-specific recommendations. Comparison with the EARS-Net data should also be made cautiously, as our study also included invasive and non-invasive sample types, while the EARS-Net data are derived from invasive samples only. Notwithstanding these limitations, several key observations may be derived: overall, the proportion of isolates with any level of acquired resistance was elevated from 44.7% between June 2014 and July 2019 to 61.9% in the recent study (2019–2023), but there was no substantial change in the proportion of MDR “ESKAPE” pathogens (23.8% vs. 23.5%) in the same periods.

Despite these constraints, this study also possesses important strengths. It focuses exclusively on data from the local ED—a clinical setting frequently underrepresented in the literature of infectious disease epidemiology, despite its critical role in initial antimicrobial decision-making. Moreover, as this research represents a second five-year surveillance cycle, it offers a perspective on local resistance dynamics that may help inform and evaluate future ASP initiatives. Finally, the integration of the WHO “AWaRe” framework enhances the practical utility of our antibiograms. This approach facilitates strategic resource allocation and aligns local clinical practice with international benchmarks for optimized antibiotic consumption, as established by the WHO and EU recommendations.

## 4. Materials and Methods

### 4.1. Study Design and Setting

A retrospective, observational study was carried out at the central ED of the Albert Szent-Gyorgyi Health Centre, University of Szeged, corresponding to the 5-year period between 1 January 2019 and 31 December 2023. This ED functions as a level I treatment center in Southern Hungary, with an annual census of 40,000 patient visits.

### 4.2. Operational Definition and Selection of ESKAPE Pathogens

In this study, “ESKAPE” pathogens were operationally defined as clinically relevant, non-duplicate isolates of *Enterococcus faecalis*, *E. faecium*, *Staphylococcus aureus*, Klebsiella spp., *Acinetobacter baumannii*, *Pseudomonas aeruginosa*, and other relevant members of the order Enterobacterales. While the original (2008) [50] designation of “ESKAPE” included *Enterococcus faecium*, *Staphylococcus aureus*, *Klebsiella pneumoniae*, *Acinetobacter baumannii*, *Pseudomonas aeruginosa*, and *Enterobacter* spp., in recent literature, this group of critical pathogens has been operationalized more broadly in a number of different ways, such as to include *Enterococcus* spp., *Klebsiella* spp., the entire Enterobacterales group and *Stenotrophomonas maltophilia* [37,51,52]. Throughout this manuscript, the term “ESKAPE pathogens” (or “ESKAPE group”) refers to this defined, extended operational panel rather than the original 2008 six-species definition.

### 4.3. Data Collection Procedures, Exclusion Criteria

This retrospective observational study included a complete census of microbiological specimens collected from patients presenting to the emergency department between 1 January 2019 and 31 December 2023. During data collection from the records of the MedBakter laboratory information system (LIS), contaminants, non-clinical (screening) samples and samples containing fungal isolates were excluded. Stool specimens were systematically excluded. Also, non-ESKAPE isolates (isolates other than “ESKAPE” pathogens listed above) were excluded.

To eliminate duplicate testing bias and prevent overestimation of pathogen prevalence, dataset deduplication was performed by retaining only the first isolate per patient per phenotype for each specific pathogen species across the entire study period; all subsequent isolates from the same patient with the same phenotype were excluded. Finally, *Stenotrophomonas maltophilia* was excluded from the final dataset due to an insufficient sample size (<30 isolates).

### 4.4. Laboratory Procedures: Microbial Identification and Susceptibility Testing

As this work represents a continuation of the institutional antibiotic stewardship program, the core procedures for bacterial isolation and identification remained unchanged, as described previously [37]. Gram-staining was carried out in the case of blood culture, respiratory tract, cerebrospinal fluid samples and other invasive sample types, to determine the presence of microorganisms and white blood cells (where relevant). The processing of urine samples was as follows: 10 µL of each un-centrifuged urine sample was cultured on UriSelect chromogenic agar plates (Bio-Rad, Berkeley, CA, USA) with a calibrated loop, according to the manufacturer’s instructions, and incubated at 37 °C for 24–48 h, aerobically. In case of the detection of relevant pathogens in a significant colony count (≥10^5^ colony-forming units/mL; however, this was subject to the interpretation of the clinical microbiologist), the plates were passed on for further processing. Aerobic and anaerobic blood culture bottles were incubated in the BacT/ALERT 3D (bioMérieux, Marcy-l’Étoile, France) detection system; blood culture bottles were incubated for 5 days (21 days, if endocarditis was suspected). Positive blood cultures, respiratory tract samples, cerebrospinal fluid samples, and other invasive sample types were routinely processed on blood agar, chocolate agar, eosine-methylene blue agar and Sabouraud agar (purchased from bioMérieux, Marcy-l’Étoile, France); bacterial culture media were incubated at 37 °C for 24–48 h, aerobically. Positive blood cultures, cerebrospinal fluid samples and other invasive sample types were also processed in Brain Heart Infusion broth (Oxoid, Basingstoke, UK) and plated on Schaedler agar (bioMérieux, Marcy-l’Étoile, France) containing horse blood 5% *v*/*v*, hemin, and vitamin K_1_, for the isolation of anaerobic organisms (not relevant to the purposes of this analysis).

Identification of bacterial pathogens was carried out using presumptive biochemical methods and matrix-assisted laser desorption/ionization time-of-flight mass spectrometry (MALDI-TOF MS). The Bruker Microflex MALDI Biotyper platform was used throughout the study period. Briefly, after cultivation of the isolates to be identified on appropriate culture media, bacterial cells were transferred to a stainless-steel target with a sterile toothpick. Following this, an on-target extraction was performed by adding 1 µL of 70% formic acid; after drying at ambient temperature, the cells were covered with 1 µL matrix (α-cyano-4-hydroxy cinnamic acid in 50% acetonitrile/2.5% trifluoro-acetic acid). MS analyses were carried out using a Microflex MALDI Biotyper (Bruker Daltonics, Bremen, Germany) in positive linear mode across the *m*/*z* range of 2 to 20 kDa; for each spectrum, 240 laser shots at 60 Hz in groups of 40 shots per sampling area were collected. For spectral analysis, the MALDI Biotyper software and MALDI Biotyper reference library (Bruker Daltonics, Bremen, Germany) were utilized. Interpretation of the results was based on the log score values (ranging between 0 and 3.0) determined by the software—by automatically calculating the proportion of matching peaks and peak intensities between the test spectrum and the reference spectra available in the database—as follows: log score values 0–1.699: “not reliable identification”, 1.70–2.299: “probable genus-level identification”, ≥2.300: reliable species-level identification.

Antimicrobial susceptibility testing (AST) was performed according to routine institutional guidance and the methodological recommendations of the European Committee on Antimicrobial Susceptibility Testing (EUCAST), using the clinical breakpoints valid in the respective year: version 9.0 in 2019, version 10.0 in 2020, version 11.0 in 2021, version 12.0 in 2022, and version 13.1 in 2023 [48]. Disk diffusion and E-tests (Liofilchem, Roseto degli Abruzzi, Italy) were carried out on Mueller–Hinton agar plates. In addition, the VITEK 2 Compact ID/AST system (bioMérieux, Marcy-l’Étoile, France) was used for verification of discrepant results. Colistin susceptibility was determined by broth microdilution using a commercial MIC panel (MERLIN Diagnostika, Bornheim, Germany). Phenotypic detection and differentiation of ESBL and AmpC production were performed using a MAST four-disc ESBL/AmpC differentiation test. Suspected carbapenemase production was confirmed using the CORIS RESIST-5 O.K.N.V.I. immunochromatographic assay. Vancomycin-resistant enterococci were screened using chromID VRE chromogenic agar (bioMérieux, Marcy-l’Étoile, France) in conjunction with routine susceptibility testing. Phenotypic detection of MRSA was performed using MRSASelect II chromogenic agar (Bio-Rad Laboratories) and cefoxitin disks (<22 mm zone diameter), and the PBP2′ Latex Agglutination Test Kit (Thermo Fisher Scientific, Waltham, MA, USA). If relevant, genotypic characterization and confirmation of resistance determinants were performed by the National Institute of Public Health and Pharmacy on a case-by-case basis. *S. aureus* ATCC 29213, *E. faecalis* ATCC 29212, *P. mirabilis* ATCC 35659, *E. coli* ATCC 25922, *P. aeruginosa* ATCC 27853, *A. baumannii* ATCC 19606 and *S. maltophilia* ATCC 13637 were used as quality control strains.

To align with the current EUCAST susceptibility category definitions and clinical breakpoint updates, the previously used “Intermediate” (I) category (that was considered “non-susceptible”) is now strictly defined as “Susceptible, increased exposure”, emphasizing that success is likely if the dosing regimen is optimized to increase drug concentration at the site of infection. AST results were interpreted according to the EUCAST breakpoint tables valid at the time of testing; therefore, after the 2019 EUCAST terminology change, isolates categorized as “I” (“Susceptible, increased exposure”) were included in the susceptible category during cumulative susceptibility analysis [49].

For each species–antimicrobial combination, susceptibility percentages were calculated using only isolates for which a valid and interpretable AST result was available for the respective antimicrobial. Isolates for which the antimicrobial was not tested, or for which no valid result was available, were excluded from the denominator for that specific species–antimicrobial combination. Missing or untested results were not imputed and were not classified as either susceptible or resistant. Selective reporting and cascade testing rules were applied according to local laboratory standards and EUCAST guidelines (e.g., secondary/reserve agents were suppressed or tested secondarily depending on primary agent resistance).

Following the results of AST, isolates were categorized based on their resistance profiles, according to the definitions used in our previous work, taking into consideration recommendations put forth by the EUCAST Expert Rules on Intrinsic Resistance, including the interpretative criteria for expected susceptible/resistance phenotypes, and acquired resistance. Antibiotics that were not tested due to intrinsic resistance or other methodological reasons were displayed as “-” in the cumulative antibiograms. Interpretation of Wt/UDR distribution in species with inducible chromosomal AmpC β-lactamases (e.g., *Enterobacter* spp., *Citrobacter* spp., *Serratia* spp. and *Morganella* spp.) should be made cautiously, as intrinsic resistance mechanisms may influence baseline susceptibility profiles and consequently the categorization of isolates, as having usual drug resistance (UDR) if resistant to at least one agent for which intrinsic resistance was not present. Multi-drug-resistant (MDR), extensively drug-resistant (XDR), and pan-drug-resistant (PDR) statuses were assigned, based on the joint ECDC/CDC expert proposal [53]. Additionally, the difficult-to-treat resistance (DTR) category was applied to Gram-negative isolates, defined as resistance to all first-line agents, including all beta-lactams (carbapenems and beta-lactamase inhibitor combinations) and fluoroquinolones [54]. As a further methodological enhancement, we integrated the WHO’s AWaRe framework into our analysis and its classification based on their potential to select for the development of bacterial resistance. Originally introduced in 2017 to monitor and optimize global antibiotic consumption, the AWaRe classification serves as a “traffic light” system to prioritize agents based on their therapeutic value and resistance development potential. “Access” group antibiotics include first- and second-line agents for infections with lower resistance potential. The “Watch” group comprises antibiotics with a higher propensity for resistance development or limited safety profiles, which should be prioritized for specific clinical indications. Finally, the “Reserve” group is restricted to “last resort” antibiotics, whose use must be strictly limited to cases where all other options have failed. Incorporating the AWaRe framework allows for a broader application of our study findings. Beyond the evidence-based foundation provided by the cumulative antibiogram for updating local empiric guidelines, the integration of this framework enables us to explicitly incorporate the “Access” group target into our institutional policies.

### 4.5. Statistical Analyses

Descriptive statistical analysis (including means or medians with ranges and percentages to characterize data) was performed using Microsoft Excel (version 16.89.1, Redmond, WA, USA, Microsoft Corp.). R software (version 4.4.3, R Foundation for Statistical Computing Vienna, Austria) was used for data analysis and visualization. Proportions are reported with 95% confidence intervals calculated using the Wilson score method.

### 4.6. Ethical Considerations

The study was conducted in accordance with the Declaration of Helsinki (1975, last revised in 2024), and national and institutional ethical standards. The ethical approval for this study was obtained from the Human Institutional and Regional Biomedical Research Ethics Committee at the University of Szeged, Hungary (reference number: 219/2018; approval date: 10 December 2018), as a part of the ongoing Antimicrobial Stewardship Program of the University of Szeged.

### 4.7. Informed Consent

This study followed established ethical guidelines for data handling, storage, and reporting to ensure patient confidentiality. Data received and used by the researchers were de-identified, per de-identification standards; therefore, information could not be traced back to patients/individuals treated at the healthcare facility. Informed consent was waived by the Institutional Review Board (IRB) as the study involved retrospective data collection without direct patient contact or identifiable information.

### 4.8. Reporting Guidelines

The present manuscript adheres to the STROBE (Strengthening the Reporting of Observational Studies in Epidemiology) reporting guidelines for observational studies, with the aim of ensuring methodological rigor, transparency, and reproducibility [55].

## 5. Conclusions

In conclusion, our data partly aligned with the European and national trends but highlighted important local differences. Our key takeaways show that “Access” antibiotics may provide the backbone of non-severe infection management, but for severe infections, the empiric treatment strategy should lean toward “Watch” antibiotics with carbapenem-sparing options in most cases. Generally, resistance to “Reserve” antibiotics remains scarce; however, as cascade testing rules restricted these agents to a subset of isolates, these findings should be interpreted within that context. This five-year study provided the necessary background data to revise and update the local antibiogram of the ED, aiding antimicrobial decision-making. By contributing to the limited body of ED-focused research on local AMR patterns using cumulative antibiograms, this study reinforces the necessity of unit-based surveillance in high-acuity clinical settings to ensure optimal empiric antibiotic use.

## Figures and Tables

**Table 1:** Total number and distribution of isolated “ESKAPE” microorganisms, 2019–2023.

Gram Stain		Species	Frequency (N)	(%)
**Gram-positive**	E	*Enterococcus faecalis*	662	10.2
		*Enterococcus faecium*	100	1.5
	S	*Staphylococcus aureus*	517	7.9
	Total (Gram-positive)		**1279**	**19.6**
**Gram-negative**	K	*Klebsiella aerogenes*	46	0.7
		*Klebsiella oxytoca*	110	1.7
		*Klebsiella pneumoniae*	715	11.0
		*Klebsiella variicola*	58	0.9
	A	*Acinetobacter baumannii*	41	0.6
	P	*Pseudomonas aeruginosa*	361	5.5
	E	Enterobacterales: *Citrobacter freundii*	40	0.6
		Enterobacterales: *Citrobacter koseri*	42	0.6
		Enterobacterales: *Enterobacter cloacae*	126	1.9
		Enterobacterales: *Escherichia coli*	2717	41.7
		Enterobacterales: *Morganella morganii*	108	1.7
		Enterobacterales: *Proteus mirabilis*	696	10.7
		Enterobacterales: *Proteus vulgaris*	32	0.5
		Enterobacterales: *Providencia stuartii*	65	1.0
		Enterobacterales: *Salmonella enteritidis*	39	0.6
		Enterobacterales: *Serratia marcescens*	35	0.5
	**Total (Gram-negative)**		**5231**	**80.4**
**Total**			**6510**	**100**

**Table 2:** Antibacterial resistance levels in “ESKAPE” bacteria, 2019–2023. Wt: wild-type, UDR: usual drug resistance, MDR: multi-drug resistance, DTR: difficult-to-treat resistance, XDR: extensive drug resistance, PDR: pan-drug resistance, CI: confidence interval.

		All (N)	Wt			UDR			MDR			DTR			XDR			N (PDR)
			N (Wt)	%	95% CI	N (UDR)	%	95% CI	N (MDR)	%	95% CI	N (DTR)	%	95% CI	N (XDR)	%	95% CI	
E	*Enterococcus faecalis*	662	380	57.40	53.60–61.12	282	42.60	38.88–46.40	5	0.76	0.32–1.76				0	0.00	-	0
	*Enterococcus faecium*	100	3	3.00	1.03–8.45	97	97.00	91.55–98.97	75	75.00	65.70–82.45				0	0.00	-	0
S	*Staphylococcus aureus*	517	325	62.86	58.62–66.92	192	37.14	33.08–41.38	64	12.38	9.81–15.50				0	0.00	-	0
K	*Klebsiella aerogenes*	46	0	0.00	-	46	100.00	92.29–100.00	17	36.96	24.52–51.40	0	0.00	-	0	0.00	-	0
	*Klebsiella oxytoca*	110	0	0.00	-	110	100.00	96.63–100.00	6	5.45	2.52–11.39	0	0.00	-	0	0.00	-	0
	*Klebsiella pneumoniae*	715	1	0.14	0.02–0.79	714	99.86	99.21–99.98	209	29.23	26.02–32.67	1	0.14	0.02–0.79	3	0.42	0.14–1.23	0
	*Klebsiella variicola*	58	0	0.00	-	58	100.00	93.79–100.00	3	5.17	1.77–14.14	0	0.00	-	0	0.00	-	0
A	*Acinetobacter baumannii*	41	7	17.07	8.53–31.26	34	82.93	68.74–91.47	29	70.73	55.52–82.39	23	56.10	41.04–70.11	2	4.88	1.35–16.14	0
P	*Pseudomonas aeruginosa*	361	242	67.04	62.03–71.68	119	32.96	28.32–37.97	34	9.42	6.82–12.87	8	2.22	1.13–4.31	0	0.00	-	0
E	*Citrobacter freundii*	40	0	0.00	-	40	100.00	91.24–100.00	14	35.00	22.13–50.49	0	0.00	-	0	0.00	-	0
	*Citrobacter koseri*	42	0	0.00	-	42	100.00	91.62–100.00	8	19.05	9.98–33.30	0	0.00	-	0	0.00	-	0
	*Enterobacter cloacae*	126	0	0.00	-	126	100.00	97.04–100.00	55	43.65	35.31–52.37	0	0.00	-	0	0.00	-	0
	*Escherichia coli*	2717	1271	46.78	44.91–48.66	1446	53.22	51.34–55.09	553	20.35	18.88–21.91	0	0.00	-	0	0.00	-	0
	*Morganella morganii*	108	0	0.00	-	108	100.00	96.57–100.00	86	79.63	71.08–86.15	0	0.00	-	0	0.00	-	0
	*Proteus mirabilis*	696	217	31.18	27.85–34.71	479	68.82	65.29–72.15	281	40.37	36.79–44.06	0	0.00	-	0	0.00	-	0
	*Proteus vulgaris*	32	1	3.13	0.55–15.74	31	96.88	84.26–99.45	10	31.25	17.95–48.57	0	0.00	-	0	0.00	-	0
	*Providencia stuartii*	65	0	0.00	-	65	100.00	94.42–100.00	51	78.46	67.03–86.71	0	0.00	-	0	0.00	-	0
	*Salmonella* spp.	39	32	82.05	67.33–91.02	7	17.95	8.98–32.67	1	2.56	0.45–13.18	0	0.00	-	0	0.00	-	0
	*Serratia marcescens*	35	0	0.00	-	35	100.00	90.11–100.00	27	77.14	60.98–87.93	0	0.00	-	0	0.00	-	0
**Total**		**6510**	**2479**	**38.08**	**36.91–39.27**	**4031**	**61.92**	**60.73–63.09**	**1514**	**23.26**	**22.25–24.30**	**32**	**0.49**	**0.35–0.69**	**5**	**0.08**	**0.03–0.18**	**0**

**Table 3:** Rate (%) of susceptibility of “ESKAPE” isolates to tested antibiotics in the WHO AWaRe “Access” group (2019–2023).

		Oxacillin	Ampicillin	Amoxicillin	Amoxicillin/Clavulanic Acid	Cefazolin	Amikacin	Gentamicin	Doxycyclin	Clindamycin	Nitrofurantoin	Sulfamethoxazole/Trimethoprim	Tetracyclin
E	*Enterococcus faecalis*	−	100.0	100.0	100.0	−	−	66.6	−	−	99.3	−	−
	*Enterococcus faecium*	−	−	3.0	3.0	−	−	57.9	−	−	−	−	−
S	*Staphylococcus aureus*	87.6	−	−	87.6	87.6	95.7	98.1	99.6	75.8	−	98.8	90.3
	MSSA				100.0	100.0	98.5	99.1	99.5	82.8		98.7	91.4
	MRSA				0.0	0.0	76.6	90.6	100.0	75.8		100.0	82.8
K	*Klebsiella aerogenes*	−	−	−	−	−	16/16	100.0	−	−	−	100.0	−
	*Klebsiella oxytoca*	−	−	−	92.7	−	96.4	98.2	−	−	−	97.2	−
	*Klebsiella pneumoniae*	−	−	−	70.7	−	97.3	86.7	−	−	−	70.8	−
	*Klebsiella variicola*	−	−	−	89.7	−	25/25	98.3	−	−	−	94.8	−
A	*Acinetobacter baumannii*	−	−	−	−	−	39.0	34.1	−	−	−	22.0	−
P	*Pseudomonas aeruginosa*	−	−	−	−	−	97.5	85.5	−	−	−	−	−
E	*Citrobacter freundii*	−	−	−	−	−	100.0	97.5	−	−	−	92.5	−
	*Citrobacter koseri*	−	−	−	78.6	−	13/13	100.0	−	−	−	95.1	−
	*Enterobacter cloacae*	−	−	−	−	−	98.5	95.2	−	−	−	93.7	−
	*Escherichia coli*	−	54.7	−	83.0	−	96.5	93.1	−	−	97.8	75.3	−
	*Morganella morganii*	−	−	−	−	−	98.3	79.6	−	−	−	66.4	−
	*Proteus mirabilis*	−	43.9	−	66.2	−	85.0	73.8	−	−	−	39.5	−
	*Proteus vulgaris*	−	−	−	71.9	−	19/19	96.9	−	−	−	87.5	−
	*Providencia stuartii*	−	−	−	−	−	23/23	93.8	−	−	−	50.8	−
	*Salmonella* spp.	−	94.7	−	97.4	−	13/13	16/16	−	−	−	100.0	−
	*Serratia marcescens*	−	−	−	−	−	26/26	100.0	−		−	97.1	−

The color coding represents the empirical utility of the given antibiotics. Green (susceptibility rate between 90 and 100%): may be considered for empirical treatment when clinically appropriate even in severe infection. Yellow (susceptibility rate between 80 and 90%): may be considered for empirical treatment when clinically appropriate except for severe infections. Red (susceptibility rate below 80%): not recommended as empirical treatment choice. Only applicable for targeted treatment options. MSSA: methicillin-susceptible *S. aureus*; MRSA: methicillin-resistant *S. aureus.* Antibiotics that were not tested due to intrinsic resistance or other methodological reasons were displayed as “−”.

**Table 4:** Rate (%) of susceptibility of “ESKAPE” isolates to tested antibiotics in the WHO AWaRe “Watch” group (2019–2023).

		Azithromycin	Clarithromycin	Erythromycin	Teicoplanin	Vancomycin	Ciprofloxacin	Moxifloxacin	Ofloxacin	Levofloxacin	Fosfomycin	Fusidic Acid	Tobramycin
E	*Enterococcus faecalis*	−	−	−	99.7	99.5	62.8	−	−	62.8	−	−	−
	*Enterococcus faecium*	−	−	−	77.0	67.0	0.0	−	−	0.0	−	−	−
S	*Staphylococcus aureus*	74.7	74.7	74.7	100.0	100.0	89.2	89.4	88.2	89.2	−	−	95.2
	MSSA	81.5	81.5	81.5	100.0	100.0	96.0	96.0	96.3	96.0	−	−	97.8
	MRSA	26.6	26.6	26.6	100.0	100.0	40.6	42.2	37.2	40.6	−	100.0	76.6
K	*Klebsiella aerogenes*	−	−	−	−	−	97.8	16/16	−	16/16	−	−	16/16
	*Klebsiella oxytoca*	−	−	−	−	−	100.0	100.0	−	100.0	−	−	96.6
	*Klebsiella pneumoniae*	−	−	−	−	−	72.7	76.9	−	76.8	51.2	−	84.3
	*Klebsiella variicola*	−	−	−	−	−	100.0	25/25	−	25/25	−	−	25/25
A	*Acinetobacter baumannii*	−	−	−	−	−	17.1	−	−	17.1	−	−	36.6
P	*Pseudomonas aeruginosa*	−	−	−	−	−	87.0	−	−	80.1	−	−	95.6
E	*Citrobacter freundii*	−	−	−	−	−	97.5	100.0	−	100.0	−	−	100.0
	*Citrobacter koseri*	−	−	−	−	−	100.0	14/14	−	14/14	−	−	14/14
	*Enterobacter cloacae*	−	−	−	−	−	92.9	95.6	−	95.6	−	−	97.1
	*Escherichia coli*	−	−	−	−	−	77.5	77.3	−	77.3	97.6	−	89.4
	*Morganella morganii*	−	−	−	−	−	84.3	87.3	−	87.3	−	−	88.9
	*Proteus mirabilis*	−	−	−	−	−	59.0	62.7	−	63.2	17.5	−	73.0
	*Proteus vulgaris*	−	−	−	−	−	96.9	22/22	−	22/22	−	−	22/22
	*Providencia stuartii*	−	−	−	−	−	64.6	16/24	−	16/24	−	−	19/22
	*Salmonella* spp.	−	−	−	−	−	87.2	18/20	−	18/20	−	−	13/13
	*Serratia marcescens*	−	−	−	−	−	97.1	26/27	−	26/27	−	−	24/24
		Piperacillin/Tazobactam	Cefuroxim	Cefoxitin	Cefixim	Cefotaxim	Ceftazidim	Ceftriaxon	Cefepime	Ertapenem	Imipenem	Meropenem	Rifampicin
E	*Enterococcus faecalis*	100.0	−	−	−	−	−	−	−	−	100.0	−	−
	*Enterococcus faecium*	3.0	−	−	−	−	−	−	−	−	3.0	−	−
S	*Staphylococcus aureus*	−	−	87.6	−	−	−	−	−	−	−	−	98.4
	MSSA	−	−	100.0	−	−	−	−	−	−	−	−	99.6
	MRSA	−	−	0.0	−	−	−	−	−	−	−	−	98.4
K	*Klebsiella aerogenes*	13/16	10/24	−	66.7	14/17	73.9	73.9	14/16	100.0	100.0	100.0	−
	*Klebsiella oxytoca*	96.6	92.1	−	96.1	96.6	96.4	96.4	96.6	100.0	100.0	100.0	−
	*Klebsiella pneumoniae*	76.3	71.5	−	72.4	75.9	73.9	73.9	76.9	99.7	99.9	99.9	−
	*Klebsiella variicola*	25/25	95.1	−	93.9	25/25	96.6	96.6	25/25	100.0	100.0	100.0	−
A	*Acinetobacter baumannii*	−	−	−	−	−	−	−	−	−	43.9	43.9	−
P	*Pseudomonas aeruginosa*	87.5	−	−	−	−	89.5	−	91.4	−	87.3	87.3	−
E	*Citrobacter freundii*	78.1	5/13	−	7/9	77.4	77.5	77.5	77.4	95.0	95.0	97.5	−
	*Citrobacter koseri*	14/14	13/21	−	26/28	14/14	97.6	97.6	14/14	100.0	100.0	100.0	−
	*Enterobacter cloacae*	85.3	26.9	−	69.0	85.3	78.6	78.6	85.3	99.2	99.2	100.0	−
	*Escherichia coli*	86.4	87.0	−	88.5	86.5	88.0	88.0	86.6	100.0	100.0	100.0	−
	*Morganella morganii*	82.5	−	−	77.8	84.1	81.5	82.4	84.1	100.0	91.8	100.0	−
	*Proteus mirabilis*	72.0	67.9	−	67.1	72.0	69.4	69.4	72.0	100.0	17/17	100.0	−
	*Proteus vulgaris*	22/22	−	−	8/10	22/22	93.8	93.8	22/22	100.0	13/13	100.0	−
	*Providencia stuartii*	24/24	21.3	−	92.7	24/24	95.4	95.4	24/24	100.0	100.0	100.0	−
	*Salmonella* spp.	24/25	5/5	−	18/18	24/25	96.7	97.4	24/25	100.0	100.0	100.0	−
	*Serratia marcescens*	26/27	−	−	8/8	27/27	100.0	100.0	27/27	100.0	100.0	100.0	−

The color coding represents the empirical utility of the given antibiotics. Green (susceptibility rate between 90 and 100%): may be considered for empirical treatment when clinically appropriate even in severe infection. Yellow (susceptibility rate between 80 and 90%): may be considered for empirical treatment when clinically appropriate except for severe infections. Red (susceptibility rate below 80%): not recommended as empirical treatment choice. MSSA: methicillin-susceptible *S. aureus*; MRSA: methicillin-resistant *S. aureus.* Antibiotics that were not tested due to intrinsic resistance or other methodological reasons were displayed as “−”.

**Table 5:** Rate (%) of susceptibility of ESKAPE isolates to tested antibiotics in the WHO AWaRe “Reserve” group (2019–2023).

		Cefiderocol	Ceftazidime/Avibactam	Ceftolozan/ Tazobactam	Colistin	Linezolid	Quinpristin-Dalfopristin	Tigecycline
E	*Enterococcus faecalis*	−	−	−	−	18/18	−	100.0
	*Enterococcus faecium*	−	−	−	−	100.0	94.5	100.0
S	*Staphylococcus aureus*	−	−	−	−	100.0	−	100.0
	MSSA	−	−	−	−	−	−	−
	MRSA	−	−	−	−	−	98.1	−
K	*Klebsiella aerogenes*	−	−	−	−	−	−	−
	*Klebsiella oxytoca*	−	−	−	−	−	−	−
	*Klebsiella pneumoniae*	−	97.3	98.1	19/20	−	−	−
	*Klebsiella variicola*	−	−	−	−	−	−	−
A	*Acinetobacter baumannii*	19/19	−	−	24/26	−	−	−
P	*Pseudomonas aeruginosa*	−	100.0	97.1	22/22	−	−	−
E	*Citrobacter freundii*	−	−	−	−	−	−	−
	*Citrobacter koseri*	−	−	−	−	−	−	−
	*Enterobacter cloacae*	−	−	−	−	−	−	−
	*Escherichia coli*	−	99.1	99.3	23/23	−	−	−
	*Morganella morganii*	−	−	−	−	−	−	−
	*Proteus mirabilis*	−	87.5	81.0	−	−	−	−
	*Proteus vulgaris*	−	−	−	−	−	−	−
	*Providencia stuartii*	−	−	−	−	−	−	−
	*Salmonella* spp.	−	−	−	−	−	−	−
	*Serratia marcescens*	−	−	−	−	−	−	−

The color coding represents the empirical utility of the given antibiotics. Green (susceptibility rate between 90 and 100%): may be considered for empirical treatment when clinically appropriate even in severe infection. Yellow (susceptibility rate between 80 and 90%): may be considered for empirical treatment when clinically appropriate except for severe infectionsOnly applicable for targeted treatment options. MSSA: methicillin-susceptible *S. aureus*; MRSA: methicillin-resistant *S. aureus.* Antibiotics that were not tested due to intrinsic resistance or other methodological reasons were displayed as “−”.

## Data Availability

The dataset is available from the corresponding author on reasonable request.

## Abbreviations

The following abbreviations are used in this manuscript:

AMR	Antimicrobial resistance
AWaRe	Access, Watch, Reserve classification
CI	Confidence interval
DTR	Difficult-to-treat resistance
EARS Net	European Antimicrobial Resistance Surveillance Network
ED	Emergency Department
EMA	European Medicines Agency
EUCAST	European Committee on Antimicrobial Susceptibility Testing
JAMREYE	Joint Action Antimicrobial Resistance and Healthcare-Associated Infections
MDR	Multi-drug resistance
MRSA	methicillin-resistant *Staphylococcus aureus*
MSSA	methicillin-susceptible *Staphylococcus aureus*
PDR	Pan-drug resistance
UDR	Usual drug resistance
UTI	Urinary tract infection
VRE	Vancomycin-resistant enterococci
WHO	World Health Organization
WT	Wild-type
XDR	Extensive drug resistance
